# The Effect of Smoking on Macular, Choroidal, and Retina Nerve Fiber Layer Thickness

**DOI:** 10.4274/tjo.galenos.2018.80588

**Published:** 2019-02-28

**Authors:** Kuddusi Teberik

**Affiliations:** 1 Düzce University Faculty of Medicine, Department of Ophthalmology, Düzce, Turkey

**Keywords:** Smoking, retinal thickness, choroidal thickness, spectral domain optical coherence tomography

## Abstract

**Objectives:**

This study aimed to compare the thickness of the macula, choroid, and peripapillary retina nerve fiber layer (RNFL) in smokers with those of healthy, nonsmoking individuals using spectral domain optical coherence tomography (SD-OCT).

**Materials and Methods:**

Sixty-eight healthy smokers with an average of 19.75 pack-years and 71 nonsmoker subjects (control group) were included in the study. Macular thickness, RNFL thickness, and choroidal thickness (CT) were measured by SD-OCT.

**Results:**

The mean age of the smokers was 42.76±6.97 years and that of the control group was 41.15±11.61 years (p=0.32). Inferonasal and temporal RNFL thicknesses were 121.60±27.40 μm and 69.75±9.82 μm in the smokers group and 109.05±21.71 μm and 75.95±15.01 μm in the nonsmoker group, respectively. The differences were statistically significant (p=0.003, p=0.005, respectively). Central macular thickness (CMT) was 222.97±18.95 μm and subfoveal CT was 369.52±105.36 μm in the smoker group, while these values were 222.98±17.72 μm and 347.42±104.63 μm in the nonsmoker group, respectively. There were no significant differences in these comparisons (p=0.99, p=0.49, respectively). A significant negative correlation was found between smoking exposure and nasal and temporal CT.

**Conclusion:**

The results of our study revealed that RNFL thickness was decreased but CMT and CT were not affected in healthy chronic smokers.

## Introduction

Nicotine addiction is one of the most significant and preventable health problems of our time. The World Health Organization predicts that by the year 2030, 9 million people worldwide will die annually due to cigarette smoking.^1,2^ Over 4,000 chemicals in cigarette smoke are known to affect the pulmonary and cardiovascular systems, and smoking is a well-known risk factor for atherosclerotic and thromboembolic events.^3^ Smoking is also a risk factor for the development of several ocular pathologies such as age-related macular degeneration, ischemic optic neuropathy, hypertensive retinopathy, cataract, glaucoma, thyroid orbitopathy, keratoconjunctivitis sicca, and strabismus in the offspring of smoking parents.^4,5,6^ Quantitative analysis of choroidal vasculature is necessary to understand the pathophysiology of choroidal disorders and to evaluate chorioretinal diseases. However, the mechanisms underlying the relationship between ocular vascular diseases and smoking are not yet known.^7^ One such method of analysis is the measurement of choroidal thickness (CT). CT has been shown to be affected by choroidal blood flow.^8^ Spectral-domain optical coherence tomography (SD-OCT) with special software programs (e.g., enhanced depth imaging) is an established method of measuring CT.

With these developments, researchers have the opportunity to examine the effect of cigarettes on the retinal and choroidal layers in more detail. The aim of the current study was to evaluate the effects of smoking on macular, choroidal, and retina nerve fiber layer (RNFL) thickness using SD-OCT.

## Materials and Methods

This cross-sectional study was performed in the Department of Ophthalmology of the Düzce University Faculty of Medicine (Düzce, Turkey) between December 2016 and June 2017. Approval for the study was obtained from the university ethics committee and written informed consent forms were signed by all the participants in compliance with the requirements of the Declaration of Helsinki. The study group consisted of healthy cigarette-smoking individuals who had no systemic or ocular disease (smoker group). Age- and gender-matched healthy individuals who had never smoked cigarettes formed the control group (nonsmoker group). In all participants, only the right eye was evaluated.

The criteria for inclusion in the study included spherical refraction between +1.0 and -1.0 diopters, best-corrected visual acuity of 20/20 or better, and axial length (AL) <25 mm. No evident systemic disease was detected in any of the study participants upon general physical examination and biochemical laboratory analyses. Subjects whose history or examinations revealed Alzheimer’s or neurological diseases such as multiple sclerosis were excluded. Upon ophthalmologic examination, no pathological findings (e.g., cornea opacities, cataract, glaucoma, media opacities, optic neuropathy) were detected in any of the subjects. Subjects who had high refractive errors, were under local or systemic medication, had used/been exposed to any neurotoxins or drugs, or had a history of ocular or systemic diseases were excluded from the study. In order to eliminate the potential influence of alcoholism or malnourishment, only those heavy smokers with good dietary habits who were healthy and did not use alcohol were selected for the study. All participants were prohibited from smoking for at least 8 h prior to taking measurements to avoid the acute effects of cigarette chemicals on the eye. In addition, the subjects were instructed not to consume any alcohol or caffeine for at least 12 h before measurements were taken.

### Ophthalmological Examinations

Demographic data were collected from all study participants. After performing refraction measurements, all subjects were tested for best-corrected visual acuity using the Snellen chart. Intraocular pressure was measured via Goldmann applanation tonometry (Nikon, Tokyo, Japan), followed by slit-lamp examination. Three drops of tropicamide and 2.5% phenylephrine were topically instilled at 5-min intervals and after a period of approximately 30 min, the subjects underwent binocular indirect ophthalmoscopy. Clinic ophthalmologists performed the dilated fundus examinations. AL measurements were then conducted with the Echoscan US 500 (Nidek Co. Ltd., Aichi, Japan). The same ophthalmologist carried out all the OCT scans to ensure consistency of results. These scans were conducted immediately after pupil dilation and again on the morning after the examination (between 8:00 and 9:00 am). 

The Heidelberg Spectralis (version 1.5.12.0; Heidelberg Engineering, Heidelberg, Germany) was employed to measure the macula and peripapillary RNFL (G: global, T: temporal, Ts: temporal superior, Ti: temporal inferior, N: nasal, Ns: nasal superior, Ni: nasal inferior). All SD-OCT images were obtained between 9:00 and 12:00 am. The Heidelberg Spectralis performed conventional OCT scans as well as enhanced depth imaging OCT, in which an inverted image of the choroid was obtained with the device positioned close to the eye. A 5°-30° rectangle incorporating the macula and optic nerve was divided into sections. Eye tracking was used to generate 100 average scans per section. 

Central foveal thickness and CT were measured from the horizontal section running straight through the center of the fovea using the Heidelberg Spectralis.^9^ Central foveal thickness was measured as the distance between the inner border of the hyperreflective line representing the internal limiting membrane and the inner border of the hyperreflective line representing the retinal pigment epithelium; CT was measured from the outer border of the hyperreflective line ascribed to the retinal pigment epithelium to the hyperreflective line of the inner sclera border. Seven measurements (one subfoveal, three temporal, and three nasal) were taken at 500 µm intervals up to 1500 µm via the software caliper. All participants completed the study.

### Statistical Analysis

All statistical analyses were performed with the Statistical Package for the Social Sciences (SPSS) software (v17.0 for Windows; SPSS Inc., Chicago, IL, USA). Descriptive statistics were determined as means ± standard deviations. Student’s t-test was used to compare qualitative data with normal distribution, and the Mann-Whitney U-test was used to compare parameters without normal distribution. Pearson correlation analysis was used to investigate associations between the parameters. P values <0.05 were considered statistically significant.

## Results

Evaluations were carried out on 68 eyes of 68 smokers (46 male, 22 female) and 71 eyes of 71 nonsmokers (46 male, 25 female). Median smoking exposure was 20 pack-years (range: 1-121). Table 1 shows the demographic and clinical characteristics of these groups and their statistical significance. 

There were no significant differences in age or gender between the smoker and nonsmoker groups. Mean intraocular pressure was 17.65±3.43 mmHg in the smoker group and 18.40±2.92 mmHg in the control group (p=0.40).

The mean AL in the smoker and nonsmoker groups was 22.95±0.82 mm and 22.93±1.01 mm, respectively (p=0.92). In the smoker group, RNFL thickness was 121.60±27.40 µm in the inferonasal quadrant and 69.75±9.82 µm in the temporal quadrant; these values were 109.05±21.71 µm and 75.95±15.01 µm respectively in the nonsmoker group. Intergroup differences in these two values were statistically significant (p=0.003 and p=0.005, respectively). There were no significant differences between the groups in peripapillary RNFL thickness in the other quadrants (Table 2). 

Central macular thickness (CMT) and subfoveal CT were 222.97±18.95 µm and 369.52±105.36 µm in the smoker group, compared to 222.98±17.72 µm and 347.42±104.63 µm in the nonsmoker group, respectively. Neither parameter was significantly different between the groups (p=0.99 and p=0.49, respectively).

The groups did not show any significant difference in nasal and temporal CT at 500, 1000, and 1500 microns (p>0.05) (Table 3). Table 4 shows a significant negative correlation between CT and smoking exposure; however, the degree of association was weak.

## Discussion

The aim of the present study was to assess the influences of smoking on the macula, RNFL thickness, and CT. The study found that the smoker and nonsmoker groups were not significantly different in terms of CMT and CT measurements. Except for the temporal and inferonasal quadrants, there were no statistically significant differences between the two groups in terms of RNFL thickness. 

The exact mechanisms of the effects of smoking on blood vessels have not been fully clarified and are still being investigated in several models. The vasoactive compounds found in cigarettes increase choroidal vascular resistance by causing vasospasm in the circulatory system. The resulting constriction of the posterior ciliary arteries can cause anterior ischemic neuropathy.^10,11^ Nicotine and carbon monoxide have been shown to accelerate atherosclerosis, and if the ophthalmic branch of the internal carotid artery is involved, ocular ischemic episodes such as amaurosis fugax could result.^12^ Studies have also shown cigarette smokers to have higher levels of erythrocytes, leukocytes, and plasma fibrinogen.^12,13,14^ Elevated levels of these blood and plasma components may increase the risk of thrombosis caused by hyperviscosity. Nicotine prompts vasoconstriction by stimulating alpha-adrenergic receptors, while the carbon monoxide found in cigarettes binds to hemoglobin, thus decreasing its oxygen transport capacity.^15^ Some studies focused on endothelial dysfunction and oxidative stress. Studies of endothelial dysfunction have reported evidence that the body’s production of endothelium-derived substances (e.g., nitric oxide, endothelin, angiotensin-converting enzyme, and tissue plasminogen activator) is altered after the ingestion of cigarette smoke.^16^ Free radicals that are either inhaled by the smoker or endogenously produced via xanthinoxidase, NADPH oxidase, or peroxidase enzymes initiate oxidative stress mechanisms. Oxidative stress products lead to abnormal nitric oxide activity, vasomotor dysfunction, smooth muscle proliferation, inflammatory cells, and thrombocyte activation.^17^ These effects on the circulatory system lead to ischemia and hypoxia in the tissue of cigarette smokers.

A few studies have evaluated the thickness of some retinal layers in smokers using OCT.^18,19,20,21^ Kumar et al.^18^ showed evidence that RNFL thinning increased more in smokers than in nonsmokers; however, the RNFL thickness in moderate smokers and severe smokers was not significantly different except in the nasal quadrant. Dervisoğulları et al.^19^ showed that mean RNFL was significantly thinner in the smoker group compared to a control group. They also reported that the inferior, superior, nasal, and temporal RNFL thicknesses were 123.1±26.1, 117.0±5.5, 64.9±8.6, and 63.5±6.8 µm in the smoker group and 130.8±11.8, 123.5±11.0, 72.4±9.8, and 58.4±7.4 µm in the nonsmoker group. Inferior and superior RNFL were significantly thinner in the smoker group (p=0.01 and p=0.03, respectively), while the nasal and temporal quadrants did not differ significantly (p=0.07 and p=0.96, respectively). In addition, the ganglion cell-inner plexiform layer complex was not significantly altered by chronic smoking.^19^ RNFL thinning has also been reported in chronic heavy cigarette smokers, and the explanations regarding the root of tobacco optic neuropathy (TON) should be explored in general to understand the underlying mechanisms.^20^ The association between smoking and TON development is attributed to the generation of reactive oxygen species and decreased blood flow due to the vasoconstrictive effect of nicotine.^21^ Additionally, cigarettes include cyanogen, a precursor of cyanide neurotoxin, which appears to contribute to TON.^22^

Duman et al.^23^ found that the RNFL thickness and all the layers of retina in healthy smokers and controls did not differ significantly. Demirci et al.^24^ reported that migraine patients who smoked exhibited decreased RNFL thickness when compared to both nonsmoking migraine patients and healthy controls. The mean and nasal RNFL thicknesses in the nonsmoker migraine patients were significantly lower than in the control group and the mean, nasal, and inferior RNFL thicknesses in the smoking migraine patients were significantly lower than in the control group. Moreover, the patients with and without aura did not show a significant difference in RNFL thicknesses. Smoking reduces retinal blood flow and hyperoxia autoregulation ability of retinal vessels due to the vasoconstrictive influences of nicotine and changed endothelial function.^25,26^

Pathological factors (polypoidal choroidal vasculopathy and central serous chorioretinopathy), pharmaceutical factors (intravitreal ranibizumab), age, axial length, refractive status, diurnal rhythm, and perfusion pressure are all recognized as causal factors in CT variation, although most of those affecting subfoveal CT have still not been identified.^27,28,29,30,31,32^ In the present study, there were no significant age, gender, or AL differences between the study and control groups. Previous studies have shown that the choroid is thicker in males than females.^7^ Although 67% of the smokers were male, no significant differences in CT were found between the two groups. The present study was conducted in the morning, as CT is known to progressively decrease after wakening from sleep.^32^ Dervişoğulları et al.^19^ demonstrated that chronic smoking does not significantly affect CT. In their study, the subfoveal CT was detected to be 304.0 in the smokers and 308.2 µm in the non-smokers. Sizmaz et al.^8^ reported smoking just one cigarette led to a significant decrease in CT lasting for at least 3 h although the initial CT measurements of the smokers and nonsmokers were not significantly different. The CT in the non-smokers was unchanged. 

Our data support those findings. We observed smokers and nonsmokers to have similar CT except during the acute phase. In our study, smokers were prohibited from smoking during the 8-h period before their SD-OCT test and thus, this intense reaction was not observed.

Kantarcı et al.^4^ reported that macular and choroidal thicknesses in long-term smokers were observed to be similar to those of healthy non-smokers. Ulas et al.^33^ did not observe any significant difference in retinal or choroidal thicknesses between smokers and nonsmokers. However, they noted that choroid was thicker in smokers the first 5 minutes after cigarette consumption but returned to baseline levels after 1 hour. They argued that the difference in results between the two studies could be due to different alignment and recording algorithms for OCT screening and different methods used for screening.^33^

Wimpissinger et al.^34^ reported that smokers showed significantly higher choroidal blood flow at baseline than nonsmokers on laser Doppler flowmetry. The increase in blood flow in nonsmokers due to carbogen respiration was found in comparison to the relatively stable blood flow in smokers. Besides, carbogen respiration did not make a significant difference in systemic hemodynamics, optic nerve head blood flow, retinal vessel diameters and blood gas values between groups. Though there were not many studies in this respect, the writers suggested an abnormal choroidal vascular responsiveness in the smokers.

Garhöfer et al.^35^ indicated that habitual smokers demonstrate impaired retinal vascular function. In their study, long-term smokers showed significantly reduced flicker-induced vasodilatation in major retinal veins, leading to a decreased response in retinal blood flow.^5^ Kool et al.^36^ determined that smoking caused acute hemodynamic and vascular fluctuations, but that there was no difference between the hemodynamic and vascular properties of the chronic smokers and nonsmokers. This finding concurs with that of the present study, which found CT in smokers and nonsmokers to be similar.

Correlation analysis in our study revealed a significant negative correlation between smoking exposure and CT. Sigler et al.^37^ also observed a negative correlation between them.

### Study Limitations

One limitation of the current study was the small sample size. Moreover, body mass index, systemic blood pressure, and plasma lipid level measurements were not included and this shortcoming might have affected CT. In addition, cessation of smoking 8 h before the measurement of CT might alter our results. We speculate that the choroidal vessels showed a reflexive dilation, resulting in the choroid being thicker than normal. Hence, in future studies, all of the smokers should be measured again without altering their normal smoking habits.

## Conclusion

In conclusion, the findings of the present study suggest that RNFL thickness is decreased in healthy heavy cigarette smokers, while CMT and CT are not affected. RNFL differences may be associated with endothelial dysfunction and retinal vascular reactivity caused by smoking. Further studies employing larger sample numbers are needed to identify the possible acute and chronic impacts of smoking on the thickness of the retina and choroid.

## Figures and Tables

**Table 1:**
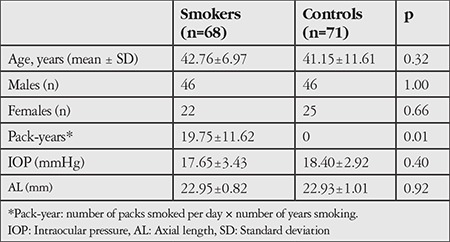
Demographic characteristics of the study participants

**Table 2:**
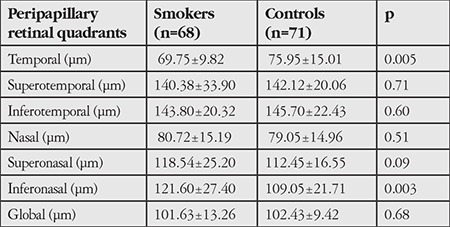
The mean peripapillary retina nerve fiber layer thickness measured by spectral domain optical coherence tomography for smokers and controls

**Table 3:**
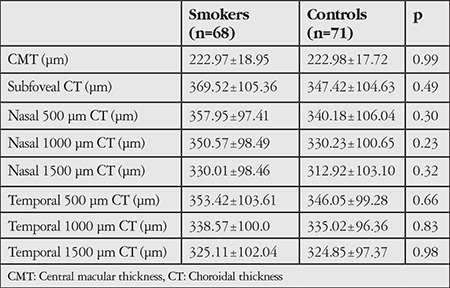
Evaluation of central macular thickness and subfoveal, nasal, and temporal choroidal thickness in the groups

**Table 4:**
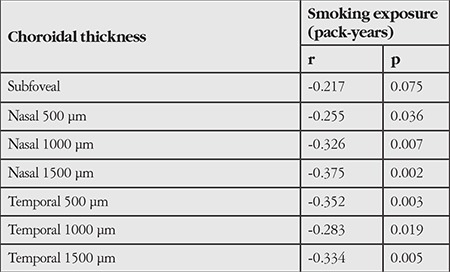
Relationship between smoking exposure and choroidal thickness
